# Anthrax in a Horse1Presented at the Fourteenth Annual Meeting of the Iowa State Veterinary Medical Association, Des Moines, Iowa, February 11 and 12, 1902.

**Published:** 1902-04

**Authors:** W. Hamilton

**Affiliations:** Boone, Iowa


### THE JOURNAL

OF

### COMPARATIVE MEDICINE AND VETERINARY ARCHIVES.

VOL. XXIII. *APRIL, 1902.* NO. 4.

### ANTHRAX IN A HORSE.[1]

BY W. HAMILTON, V.S.,
BOONE, IOWA.

THE subject of this report was a bay gelding, about seventeen years old, in good condition, weighing about 1300 pounds, and having a previous history of good health. On November 9, 1901, the horse was noticed to be a little stiff in the left hind leg, but no swelling or fever was perceptible to the owner. The appetite was good up to the morning of the 10th. At that time the owner noticed that the animal was unable to stand around in the stall, and was terribly swollen over the left hip. About the centre of the swelling was a small, round hole, about the size of a puncture made by a shingle-nail, from which was exuding a dark-colored fluid mixed with gas and having the disagreeable odor of decaying tissue.

I arrived at the place about 11 o'clock, and found the subject standing on the barn floor, with the left hind leg flexed at fetlock joint, so that the front part of the joint was nearly touching the floor. There was a twitching of the muscles back of the shoulders and in the flank of the right side, and a peculiarly anxious expression of the face. Temperature was 103° F.; pulse 70 and wiry. At intervals of from five to twenty minutes there was violent straining, as if to expel either water or feces, although there had been natural passages previous to the straining. The swelling over the left hip extended from the anterior border of the ilium to the hock joint, and was cold and insensible. I made a couple

[1] Presented at the Fourteenth Annual Meeting of the Iowa State Veterinary Medical Association, Des Moines, Iowa, February 11 and 12, 1902.

of deep incisions in the swelling, but could not discern the least evidence that this gave him any pain. The swelling resembled the swelling of blackleg in cattle, only it was more tense, and the crackling of air under the skin could not be induced by pressure, although there was gas escaping from the hole before described and from the incisions made by the knife, along with a dark red liquid, which was of a very disagreeable odor. About three o'clock in the afternoon the horse sank to the ground and lay flat upon his side most of the time, except when the spells of tenesmus would come on, when once or twice he got up on the front legs, but never made any attempt to raise himself behind. He continued about the same until seven o'clock of the 10th, when he succumbed.

There was no treatment of any kind prescribed. On the 11th I went to the place with the intention of making a careful post-mortem examination, but the smell and surroundings were almost unbearable, so I was content with a very superficial examination. The external appearance was much the same all over the body as the left hind leg had been the day previous, the swelling extending over the entire body, the skin being so tense as to cause it to rupture on the median line and around the rectum and sheath. After putting on rubber gloves I opened the body along the median line. There was considerable serum in the connective tissue. The kidneys were very soft and almost ready to break down. The spleen was very much enlarged for two-thirds of its length, and dark colored. I removed and took away with me a piece of the spleen and the liver. From the blood squeezed out of the spleen I inoculated a rabbit, which died in less than twenty-four hours; I also prepared two specimens for the microscope. These were colored with methylene blue. Both Dr. Lindahl, a physician of Boone, and myself examined them, and were satisfied that there were present plenty of anthrax bacilli.

### Discussion.

Dr. Gibson said the symptoms described were not those of anthrax, and that he could not accept the diagnosis of anthrax. At the request of a member he described briefly the symptoms of anthrax in the horse as seen by himself.

Dr. C. E. Stewart reported a very interesting experience with anthrax within the past two years. Two years ago eleven cows and twenty hogs died suddenly. On investigation he made a diagnosis of anthrax. These animals were infected by feeding in a pasture over which a cow, which had died suddenly of what he supposed was anthrax, had been dragged. He vaccinated the other animals on the farm, and the disease did not spread further. Last year a cow died of what he supposed was anthrax. The carcass was allowed to lie unburied in the pasture. Later four horses were put into the pasture. They made a resting-place of the spot where the carcass had lain. Within a short time they all became sick, showed symptoms of anthrax, and died within a day. Three dogs that had eaten of the carcasses took sick, and two of them died.

